# Health outcomes of cancer patients during the first year following admission to palliative care: A longitudinal study in Ho Chi Minh City, Vietnam

**DOI:** 10.1097/MD.0000000000049840

**Published:** 2026-07-17

**Authors:** Thuy Mai, Oanh Trinh, Dung Do, Ninh Uong, Cheng-Pei Lin, Richard Harding

**Affiliations:** a Epidemiology Department, Faculty of Public Health, University of Medicine and Pharmacy at Ho Chi Minh City, Vietnam; b Center of Clinical Biomedical Testing and Scientific and Technical Services, Pasteur Institute in Ho Chi Minh City, Vietnam; c Institute of Community Health Care, College of Nursing, National Yang Ming Chiao Tung University, Taipei, Taiwan; d Faculty of Nursing, Midwifery & Palliative Care at King’s College London, UK.

**Keywords:** APCA POS, health care, longitudinal study, outcome assessment, palliative care, quality of life

## Abstract

Palliative care can enhance health care outcomes for patients with life-threatening conditions. However, data regarding the prevalence and consequences of palliative care services for end-of-life patients in Vietnam are limited. Therefore, there is an urgent need to assess the efficacy of palliative care. This evidence supports the advancement of these services. To examine changes in mean health outcome scores, measured by the African Palliative Care Association Integrated Palliative Outcome Scale, among cancer patients receiving palliative care, and to assess their health improvements. We conducted a longitudinal study at Oncology Hospital, a tertiary center in Ho Chi Minh City, Vietnam, from July 2020 to January 2022. Patient and caregiver outcomes were measured monthly, within 3 days of admission, and continued until the patient’s death or the end of follow-up. The study included 134 newly admitted patients and their caregivers, all of whom were referred to palliative care within 3 days of admission. Notably, patients with cancer showed an overall improvement in health outcomes, as evidenced by a significant monthly reduction in scores of 4.6 points (*P* < .01). The most significant progress was observed among those with physical symptoms, who experienced an average monthly decrease of 2.7 points. Furthermore, improved patient health outcomes were associated with an initial cancer diagnosis, older age, and caregiver health status. Palliative care can improve health outcomes of patients with cancer over time. Changes in the patients’ quality of life are influenced by psychological burden, physical symptoms, initial diagnosis, patient age, and caregiver health. Understanding these changes can help palliative care healthcare professionals achieve optimal patient outcomes at any stage of the disease.

## 1. Introduction

Palliative Care (PC) refers to specialized care for individuals with diseases that cannot be cured or treated. It aims to alleviate pain and offers support during challenging times. In the context of patients with cancer, PC involves measures to improve their quality of life. These include prevention and early detection of problems, pain management, counseling for physical symptoms, and addressing psychological and social issues for both the patients and their families.^[1,2]^ Currently, the need for PC is increasing due to rising cancer incidence and is especially critical in regions where terminal cancer rates are high and cure rates are low.^[3]^

Cancer incidence and mortality are increasing in Vietnam, with 159 new cases and 106 deaths per 100,000 people.^[4]^ Most cases are detected late, with approximately 150,000 new late-stage diagnoses each year.^[5,6]^ This reduces the chances of survival, and many spend their final days in pain, anxiety, or depression. The demand for PC is high.^[7,8]^ In 2005, Vietnam had no specific PC policy. The Ministry of Health initiated a PC program for cancer patients with support from the Harvard Medical School PC Working Group, resulting in a National Guide in 2014.^[7]^ PC models are now present in several hospitals and are transitioning to home care. As demand increases, more strategies are needed to expand access to healthcare.^[8]^

A systematic review in the Asia-Pacific region found that although PC research has grown, it has primarily focused on high-income countries, leaving many low- and middle-income countries with few publications. More resources are needed to support research in these countries and develop PC services.^[9]^ In Vietnam, the healthcare system is still growing, with few studies on the effectiveness of PC in patients with cancer. This data gap hinders improvements in PC policies and patient care. There is an urgent need for research on the health outcomes of patients receiving PC to demonstrate the effectiveness of the PC model and its influencing factors. This study examined symptom improvement in cancer patients at the Oncology Hospital in Ho Chi Minh City over a 12-month period, using the African Palliative Care Association Palliative Outcome Scale (APCA POS).

## 2. Materials and methods

The study followed the STROBE reporting guidelines.^[10]^

### 2.1. Study design and setting

This longitudinal prospective study was conducted at HCMC Oncology Hospital from July 2020 to January 2022. This study examined the characteristics, risk factors, and changes in the health outcomes of 134 patients admitted to the palliative care department. Patients were referred from the Emergency Department or other related departments based on their medical records and symptoms, in accordance with Ministry of Health guidelines.^[7]^

### 2.2. Recruitment

**Figure 1.**
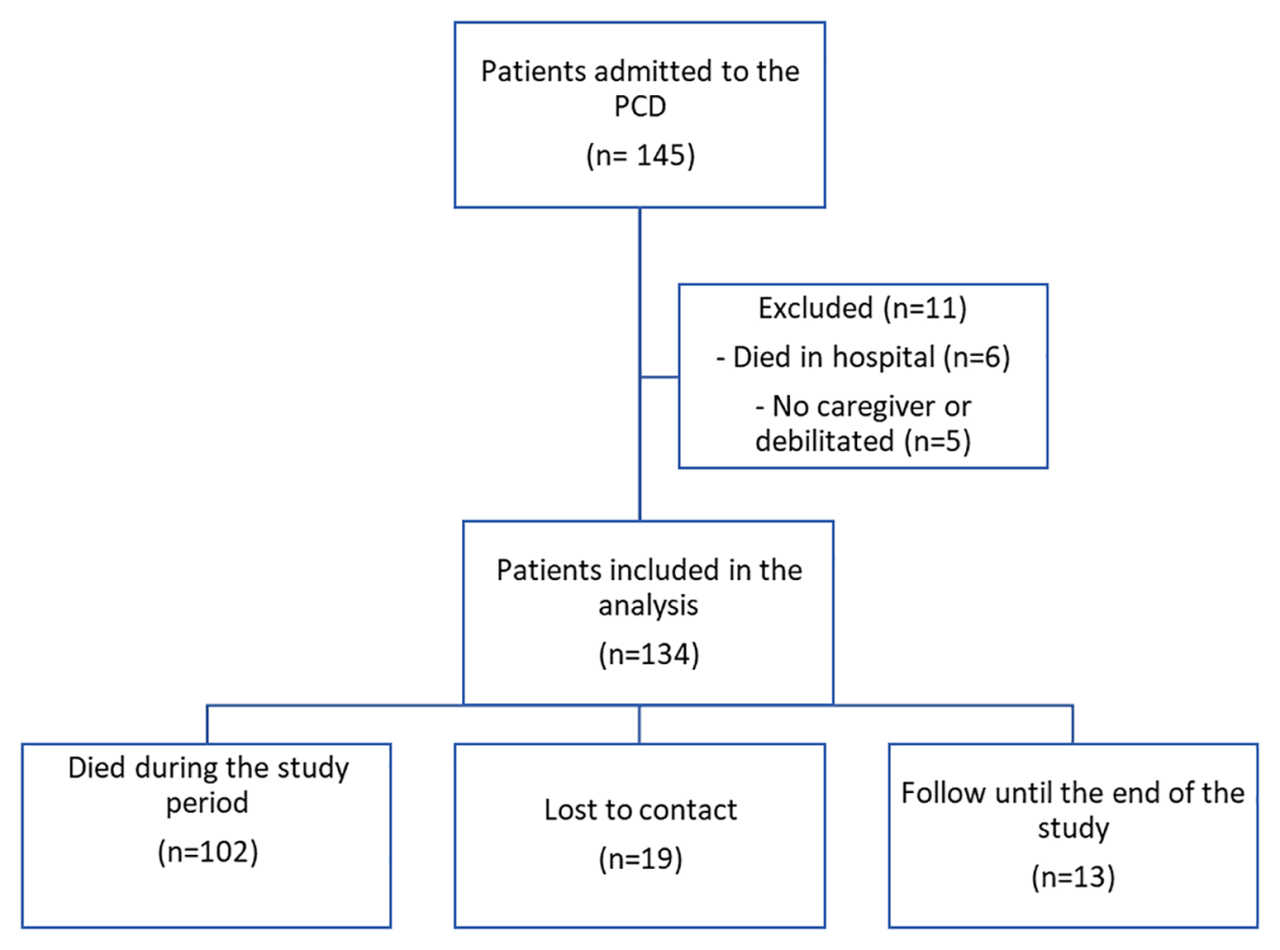
Participant flow diagram. PCD = palliative care department.

Patients were consecutively recruited from a list of daily admissions. To be eligible, they needed a referral within 3 days, to be at least 18 years old, and to have an informal caregiver. Approximately 70% of the patients had caregivers.^[11]^ Figure 1 shows a flowchart of the participant. A total of 145 patients were admitted to the palliative care department between July 2020 and January 2022. Eleven patients were excluded (five because no caregiver or debilitated, and six because they died in the hospital before completing at least 2 interviews). Thus, 134 patients with at least 2 APCA POS assessments were included in the analysis. At the end of the study, 102 patients had died (76.1%), 19 were lost to contact (14.2%), and 13 were followed until the study end (9.7%). Figure 1 shows the participant flow.

### 2.3. Data collection

Baseline data were collected directly at the hospital upon admission to the Palliative Care Unit. This included demographic and clinical variables as well as the APCA POS assessment. Follow-up data, including APCA POS periodic measurements, were collected monthly, primarily by telephone or occasionally in person when patients attended appointments. The researcher confirmed patient participation 2 weeks prior through contact with the patient or a designated person. This process was performed monthly until the patient died, could not be contacted, or the study ended. This coordinated approach was appropriate for both the baseline (in person) and follow-up (mainly by telephone) phases. However, it could introduce bias and limitations, such as under-reporting of symptoms during telephone interviews due to a lack of direct observation. This is addressed in the limitations section.

### 2.4. Measures

The health outcomes of cancer patients receiving PC were assessed using the Vietnamese version of the APCA POS.^[12]^ This questionnaire measures physical and psychological symptoms, spiritual, practical, and emotional concerns, and psychosocial needs of the patient and family.

#### 2.4.1. African palliative care association integrated palliative outcome scale (APCA POS)

The Palliative Care Outcome Scale (POS) is a set of tools used to assess physical symptoms and psychological, emotional, and supportive needs. These validated tools are applicable in clinical care, audits, research, and training. The POS has been developed for people with cancer and other chronic illnesses.^[12]^ The APCA POS builds on the first version of the POS, which is valid and reliable for measuring the health status of cancer patients receiving PC in developing countries.^[13]^ The APCA POS identifies common symptoms and concerns of terminally ill patients and their families, such as pain, anxiety, mental health, information, and family support needs. Identifying these issues facilitates timely intervention, reduces caregiver stress, and enhances the quality of life of both the patients and their families. Patients, caregivers, and healthcare professionals were able to complete the questionnaire. The tool consists of 16 items rated on a Likert scale from 0 (none) to 5 (overwhelming), with higher scores indicating greater symptom impact and unmet needs.^[14]^

#### 2.4.2. Australia-modified karnofsky performance Scale (AKPS)

The Australia-modified Karnofsky Performance Scale measures the patient’s ability to perform daily activities. It provides a score ranging from 0 to 100, with lower scores indicating greater impairments.^[15]^

#### 2.4.3. Palliative phase of illness

The Illness Status Classification is a clinician-assessed tool. It considers a patient’s and their caregivers’ symptoms, functions, and needs. The scale groups patients’ status as stable, unstable, deteriorating, impending death, or death. It is a reliable tool for planning and improving the quality of patient care.^[16]^

### 2.5. Study size

The sample size was calculated based on the changes in the APCA POS score.^[17]^ Considering the APCA POS score at admission and six follow-up assessments, which showed weak to moderate correlations between baseline and follow-up, a minimum of 134 patients was required to detect a small effect size of 0.25 in APCA POS change scores, with 80% power and a 2-sided alpha level of 0.05. It was estimated that approximately 30% of the patients would discontinue participation during the study.^[18]^

### 2.6. Statistical analysis

Descriptive statistics were performed for patient and caregiver demographics at admission and APCA POS scores at follow-up. Chi-square and Mann–Whitney *U* tests were used to compare age, sex, and APCA POS scores between participants and nonparticipants.

This study used the linear mixed-effects model (LMM) to evaluate changes in APCA POS scores over time, assuming that missing data were missing-at-random (MAR).^[19]^ However, missing data on APCA POS scores due to mortality may be missing-not-at-random (MNAR). This could lead to an inaccurate estimate of APCA POS scores, as distinguishing between MAR and MNAR missing data mechanisms is often unclear.^[20]^ The LMM was used in this study to account for changes at 2 levels: within individual patients (through repeated measurements) and between patients. The model incorporated fixed effects (to assess the overall association between time and APCA POS scores) and random effects (to account for inter-patient differences in the trends of APCA POS score changes). It assumes that each patient has a unique baseline APCA POS score and a distinct trajectory of change. The fit of the LMM was compared with that of an ordinary linear model using the likelihood ratio test. If the *P*-value was <.05, the LMM was deemed more appropriate.

To assess robustness to potential MNAR mechanisms due to high mortality, we performed a sensitivity analysis by restricting the sample to observations from month 6 onward. Additionally, exploratory quadratic mixed-effects models were fitted by adding a quadratic term for time to evaluate potential nonlinearity in the trajectories of APCA POS scores. All models were estimated using maximum likelihood.

### 2.7. Ethics approvals

Written informed consent was obtained from all participants. The study protocol was approved by the Biomedical Research Ethics Committee of Ho Chi Minh City Oncology Hospital (Reference Number: 427/BVUB-HDD) and the Research Ethics Committee of King’s College London (Reference Number: HR-18/19–10,835).

## 3. Results

### 3.1. Overview of the participants

Table 1 presents the baseline characteristics of patients admitted to the PC department during the study period, along with those of their caregivers. The mean age of the patients was 58 years. Most patients were married, and over half of them required a caregiver to complete the questionnaire on their behalf. A significant proportion of patients with PC were diagnosed with digestive organ cancer or head and neck cancer. Although 43.3% of patients were in a stable condition, more than half were in an unstable or deteriorating. The prevalence and severity of patients’ physical symptoms, as well as their psychological and emotional problems at admission, have been previously published.^[21]^

**Table 1:** Characteristics of patients and caregivers.

Characteristics of patients	n = 134	n (%)
**Female**		59 (44.0)
**Age**		58.1 ± 14.1*
**Respondents**	Patients	58 (43.3)
	Family/friend help answer	76 (56.7)
**Have financial hardship**	Yes	83 (61.9)
**Responsible for a family member or a friend**		
	An adult, 18 or over	9 (6.7)
	One or more children	18 (13.4)
	None	107 (79.9)
**Primary diagnosis**	Digestive organs	29 (21.6)
	Head—face - neck	23 (17.2)
	Respiratory and intrathoracic organs	22 (16.4)
	Breast	18 (13.4)
	Genital organs	18 (13.4)
	Other	12 (9)
	Liver	8 (6)
	Pancreatic	4 (3)
**Palliative phase of illness**	Stable	58 (43.3)
	Unstable	40 (29.8)
	Deteriorating	34 (25.4)
	Dying	2 (1.5)
**Characteristics of caregivers**	**n = 134**	**n (%**)
**Female**		95 (70.9)
**Age**		47.2 ± 13.5*
**The relationship with patients**	Spouse or partner	57 (42.6)
	Sibling	11 (8.2)
	Son or daughter	48 (35.8)
	Other (Parent, cousin, friend)	18(13.4)
**Health status**	Much less than most	28 (20.9)
	Less than most	68 (50.7)
	About the same	34 (25.4)
	More than most	4 (3)
**Average hours per day caring for the patients:**		19.9 (6.9)* 24 (12–24)†

* Mean (SD).

† Median(Quartiles).

Nearly 3-quarters of caregivers were middle-aged women, and most were married. Typically, caregivers were close family members such as spouses and children, whereas a smaller proportion were siblings or parents. Most caregivers rated their health as reasonable or fair at the time of admission. More than half of the participants reported participating in fewer social activities than the others. Additionally, almost all caregivers dedicated nearly the entire day to their patients, spending an average of 20 hours daily, even when not actively engaged in care. The results also showed that the majority of caregivers had low monthly incomes.

### 3.2. Changes in the APCA POS scores of cancer patients compared to the time of admission

**Figure 2.**
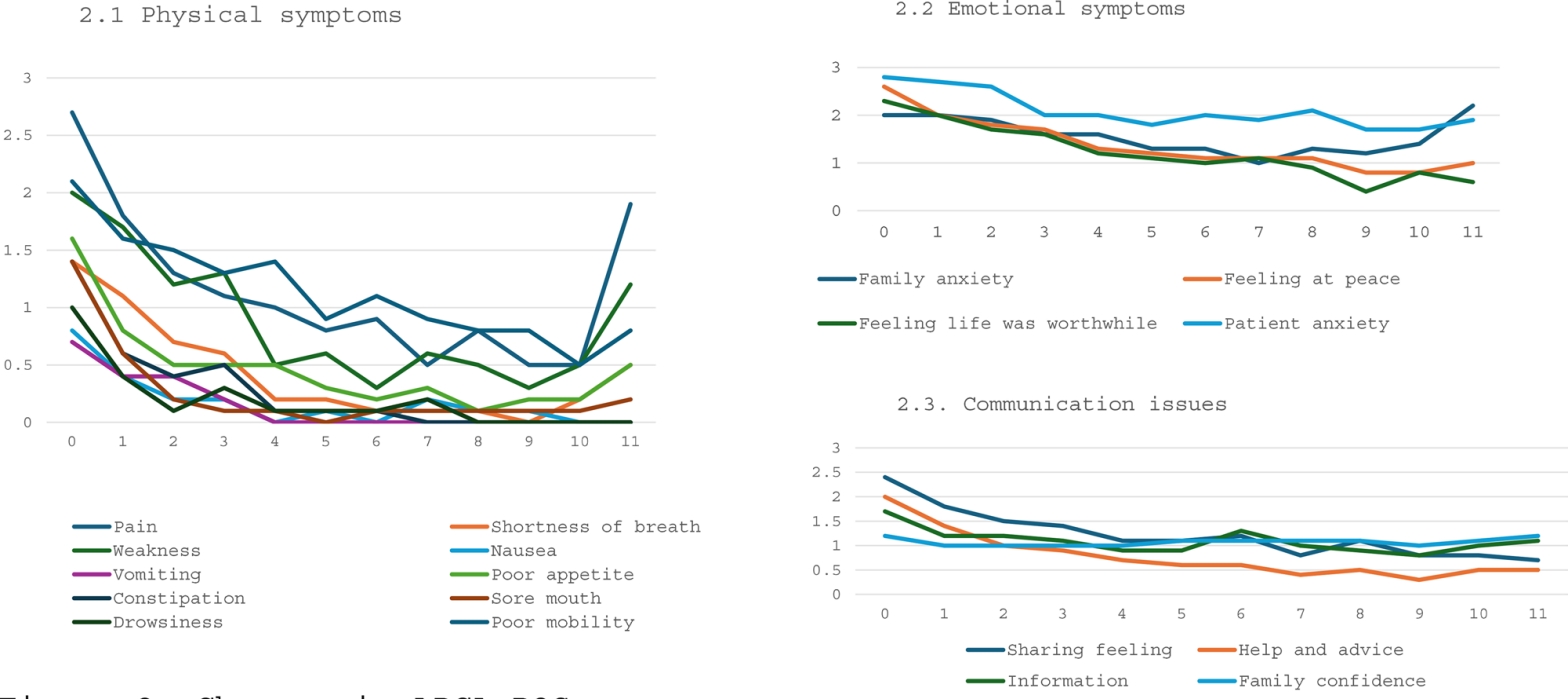
Changes in APCA POS scores over time (higher score = worst status). (Fig. 2.1) Physical symptoms. Changes in pain, shortness of breath, weakness, nausea, vomiting, poor appetite, constipation, sore mouth, drowsiness, and poor mobility. (Fig. 2.2) Emotional symptoms. Changes in family anxiety, feeling at peace, feeling life was worthwhile, and patient anxiety. (Fig. 2.3) Communication issues. Changes in sharing feeling, help and advice, information, and family confidence.

Figure 2 illustrates the changes in patients’ APCA POS scores for physical symptoms, emotions, communication, and psychosocial problems during the follow-up period, with higher scores indicating worse clinical conditions. After 7 months, the pain significantly decreased from 2.7 points to approximately 1 point, but then increased in the final month. Similar trends were observed for shortness of breath, weakness and fatigue. The remaining symptoms gradually decreased by the end of the follow-up period (Figure 2.1). Additionally, the emotional states of the patients and caregivers showed inconsistent improvements over time. Patients’ feelings that life was worthwhile improved most noticeably (from 2.3 to 0.6 points), while patient and family anxiety tended to increase in the final month (Figure 2.2). Figure 2.3 shows that over time, patients were able to share more problems and receive more support from relatives, as indicated by the gradually decreasing scores. However, there was a slight increase in scores at the end of the follow-up period.

Table 2 presents multilevel modeling assessing changes in APCA POS scores among cancer patients from the time of admission. There was a significant decline in the total APCA POS scores during the PC period. The patients’ total APCA POS scores during the follow-up period decreased significantly compared with their admission scores and with each preceding time point, particularly in the early months, with a notable reduction from 12.89 to 8.65 points (*P* < .01). The decline continued each month, dropping from 18.8 to 13.25 points compared to the first month, indicating significant improvement in the patients’ condition over time, with p-values < 0.01 at all interview points.

**Table 2:** Multilevel mixed-effects models assessing APCA POS score trajectories over time, including exploratory comparison of linear and quadratic models.

Time assessment	APCA POS total*		Physical symptoms*		Emotional symptoms*		Communication issues*	
	Coefficient (SE)	95% CI	Coefficient (SE)	95% CI	Coefficient (SE)	95% CI	Coefficient (SE)	95% CI
Admission	Ref.		Ref.		Ref.		Ref.	
1-month	−8.65 (0.61)	(–9.84, –7.45)	–5.72 (0.41)	(–6.53, −4.91)	–0.96 (0.24)	(–1.45, −0.48)	–1.96 (0.19)	(–2.33, −1.59)
2-month	−11.51 (1.00)	(–13.48, −9.53)	–6.94 (0.69)	(–8.31, −5.57)	–1.36 (0.36)	(–2.07, –0.64)	–3.00 (0.27)	(–3.54, −2.47)
3-month	−12.89 (1.51)	(–15.87, –9.92)	–6.95 (1.05)	(–9.02, –4.88)	–2.24 (0.51)	(–3.24, –1.25)	–3.38 (0.36)	(–4.10, –2.67)
4-month	−14.72 (1.98)	(–18.62, −10.83)	–7.93 (1.38)	(–10.65, –5.22)	–2.43 (0.63)	(–3.67, –1.18)	–4.11 (0.43)	(–4.96, −3.27)
5-month	−15.72 (2.51)	(–20.65, –10.80)	–8.05 (1.75)	(–11.49, –4.62)	–3.05 (0.79)	(–4.60, –1.51)	–4.15 (0.53)	(–5.19, –3.11)
6-month	−15.36 (3.01)	(–21.26, –9.46)	–7.82 (2.09)	(–11.93, –3.71)	–3.31 (0.92)	(–5.12, –1.50)	–3.57 (0.61)	(–4.77, –2.37)
7-month	–16.14 (3.50)	(–23.02, –9.26)	–7.47 (2.44)	(–12.27, –2.68)	–3.56 (1.06)	(–5.64, –1.49)	–4.33 (0.69)	(–5.68, –2.98)
8-month	−16.45 (4.03)	(–24.35, −8.54)	–7.75 (2.81)	(–13.25, –2.24)	–3.69 (1.21)	(–6.06, –1.32)	–4.05 (0.78)	(–5.59, –2.52)
9-month	−18.80 (4.56)	(–27.75, –9.84)	–8.18 (3.16)	(–14.40, –1.97)	–5.18 (1.36)	(–7.86, –2.51)	–4.47 (0.88)	(–6.20, –2.75)
10-month	−17.97 (5.09)	(–27.95, –7.99)	–7.60 (3.54)	(–14.81, –0.91)	–4.56 (1.49)	(–7.49, –1.63)	–3.88 (0.96)	(–5.77, –2.00)
11-month	−13.25 (5.56)	(–24.16, –2.33)	–4.57 (3.87)	(–12.17, 3.02)	–3.52 (1.63)	(–6.73, –0.31)	–3.82 (1.05)	(–5.88, –1.76)
**Constant**	32.24 (0.87)	(30.52, 33.97)	15.13 (0.61)	(13.92, 16.33)	9.72 (0.31)	(9.12, 10.32)	7.38 (0.28)	(6.83, 7.94)
***P*-value**	.000		.000		.000		.000	
**Random effects** †	**Estimate(SD**)	**95% CI**	**Estimate(SD**)	**95% CI**	**Estimate(SD**)	**95% CI**	**Estimate(SD**)	**95% CI**
Between-person effect	9.19 (0.67)	(7.95, 10.62)	6.46 (0.47)	(5.61, 7.46)	2.96 (0.25)	(2.51, 3.49)	2.91 (0.21)	(2.51, 3.36)
Within person effect	4.37 (0.21)	(3.97, 4.81)	2.96 (0.16)	(2.65, 3.31)	1.93 (0.10)	(1.73, 2.14)	1.50 (0.06)	(1.38, 1.63)

Exploratory comparison of linear versus quadratic mixed-effects models for total APCA POS score								
Model	Time β (SE)	Time^2^ β (SE)	LR χ^2^ vs Linear	*P*-value	Log likelihood			
Linear	−1.376 (0.171)	–	–	–	−639.30			
Quadratic	−4.297 (0.356)	+0.283 (0.032)	66.25	<.0001	−606.17			

* APCA POS score: high scores indicated worse patient status.

† *P*-value of Multilevel model < 0.001.

95% CI = 95% confidence interval, SD = standard deviation, SE = standard error.

LR χ^2^ = Likelihood Ratio Chi-square test statistic comparing nested models. The quadratic model was exploratory and performed on available data with sufficient follow-up (197 observations from 19 patients). The positive coefficient for Time^2^ indicates deceleration of improvement over time (nonlinear trajectory).

For the sub-scores of the total APCA POS score, the patients’ physical symptom scores showed the most significant decrease, from 8.18 to 4.57 points (*P* < .01). Similarly, the patients’ emotional and communication problems also improved over time, with scores decreasing from 5.18 to 0.96 points (*P* < .01).

### 3.3. Changes in the APCA POS scores of cancer patients over time and related factors

Based on the analysis of mixed-effects regression models across the entire study sample and separately for questionnaires completed by patients themselves or by proxies, there was no statistically significant difference in the change in the total score over time between the self-responding patient group and proxy-responding group. The estimated coefficients for each time level in the 2 subgroup models showed no statistically significant differences (all *P* > .05). However, there was a substantial difference at baseline, with self-reported scores lower than those reported by proxies.

The results of multilevel modeling indicated that patient health outcomes generally improved over time. The most significant improvement was observed in physical symptoms, with the mean APCA POS score decreasing significantly by 2.79 points per month of follow-up (95% CI: −3.38 to −2.2). Relationship problems and family support issues also declined significantly (β = −1.02, 95% CI: −1.26 to −0.78). Emotional and psychological symptoms gradually decreased each month (β = −0.58, 95% CI: −0.82 to **−**0.35). The analysis also showed that newly admitted patients with PC had significantly different initial health statuses. Moreover, patients with higher baseline APCA POS scores tended to show a more rapid improvement, although the extent of improvement varied across individuals.

In addition, when analyzing the caregivers’ APCA POS scores separately, aspects such as anxiety, the need for more disease-related information, and confidence in caring for patients were considered. Table 3 presents the multilevel model assessing changes in caregivers’ APCA POS scores from the time of admission. The results showed that caregivers’ APCA POS scores followed a similar improvement trend to total APCA POS scores, with reductions from month 1 to month 9followed by an upward trend in the final months of follow-up.

**Table 3:** Multilevel modeling assessing the change in APCA POS score of caregivers compared to the time of admission.

Variables	APCA POS score*		
	Coefficient (SE)	95% CI	*P*-value
**Time assessment**			
Admission	Ref.		
1-month	−0.71 (0.15)	(−1.007, −0.411)	<.001
2-month	−1.08 (0.21)	(−1.51, −0.66)	<.001
3-month	−1.72 (0.28)	(−2.28, -1.15)	<.001
4-month	−1.81 (0.34)	(−2.48, -1.14)	<.001
5-month	−1.78 (0.41)	(−2.58, -0.97)	<.001
6-month	−1.15 (0.47)	(−2.07, -0.23)	.014
7-month	−1.51 (0.52)	(−2.54, -0.47)	.004
8-month	−1.47 (0.59)	(−2.64, -0.3)	.014
9-month	−2.05 (0.66)	(−3.35, -0.74)	.002
10-month	−1.77 (0.72)	(−3.19, −0.35)	.014
11-month	−1.34 (0.78)	(−2.89, 0.19)	.087
**Constant**	5.71 (0.18)	(5.34, 6.08)	<.001
**Random effect**	**Estimate(SD**)	**95% CI**	***P*-value**
Between-person effect	1.79 (0.15)	(0.17, 0.73)	<.001
Within person effect	1.22 (0.05)	(1.11, 1.33)	

* APCA POS score: Total score of caregiver anxiety, confidence and information needs.

95%CI: 95% confidence interval, SE = standard error, SD = standard deviation.

Table 4 presents the changes in APCA POS scores after adjusting for the dependent variables. The results showed that the total APCA POS score, physical symptoms, emotional issues, and psychosocial problems in patients with cancer generally decreased over time. In particular, the total APCA POS score decreased by 4.6 points (95% CI: **−**5.49 to −3.71), physical symptoms decreased by 2.78 points (95% CI: −3.37 to **−**2.19), emotional issues decreased by 0.58 points (95% CI: −0.82 to **−**0.35), and psychosocial problems decreased by 1.03 points (95% CI: **−**1.26 to **−**0.78). Patient age, type of cancer diagnosis, and caregiver health status were all associated with changes in APCA POS scores. Patients aged 65 years and older exhibited lower emotional issues (β = **−**2.04, 95% CI: −4.1 to -0.09) than those aged 18 to 35 years. Compared with patients with gastrointestinal cancers, patients with a diagnosis of genitourinary cancers had higher APCA POS total scores (β = 5.63, 95% CI: 1.54 to 10.37) and physical symptoms (β = 1.52, 95% CI: 0.34 to 6.7). Patients with head, face, and neck cancers had increased emotional symptoms (β = 1.54, 95% CI: 0.05–3.03) and psychosocial problems (β = 1.79, 95% CI: 0.45–2.91). Respiratory cancer was associated with higher psychosocial problems (β = 1.35, 95% CI: 0.15–2.56). Caregiver health status from moderate to poor was significantly associated with a higher patient APCA POS total score (β = 3.61, 95% CI: 0.15–3.67), emotional symptoms (β = 1.34, 95% CI: 0.41–3.61), and communication problems (β = 1.13, 95% CI: 0.34–1.91), but not with physical symptoms.

**Table 4:** Multilevel modeling assessing the changes in APCA POS scores of cancer patients over time and related factors.

Variables	APCA POS total score†		Physical symptoms†			Emotional symptoms†			Communication issues†	
	Coefficient (SE)	95% CI	Coefficient (SE)	95% CI	Coefficient (SE)		95% CI	Coefficient (SE)		95% CI
Time	**−4.6 (0.45**)**	**(−5.49, −3.71**)	**−2.78 (0.3**)**	**(−3.37, −2.19**)	**−0.58 (0.11**)**		**(−0.82, −0.35**)	**−1.03 (0.12**)**		**(−1.26, −0.78**)
Age										
18-35	*Ref*									
35–64	**−**0.54 (2.74)	(**−**5.93, 4.83)	0.13 (1.97)	(**−**3.73, 4.01)	**−**1.5 (0.97)		(**−**3.43,0.42)	0.45 (0.82)		(-1.17, 2.07)
65+	**−**2(2.86)	(**−**7.62, 3.61)	**−**0.9 (2.06)	(**−**4.94, 3.13)	**−2.04 (1.01**)*		**(−4.1,-0.09**)	0.83 (0.86)		(-0.85, 2.52)
Diagnosis										
Digestive organs	*Ref*									
Respiratory and intrathoracic organs	0.6 (2.1)	(**−**3.12, **−**5.02)	0.61 (1.46)	(**−**2.26, 3.48)	**−**0.71(0.75)		(**−**2.1, 0.77)	**1.35 (0.61**)*		**(0.15, 2.56**)
Head: face - neck	3 (2.1)	(**−**0.76, **−**7.49)	**−**0.1 (1.52)	(**−**3.08, 2.87)	**1.54 (0.76**)*		**(0.05, 3.03**)	**1.79(0.63**)*		**(0.45, 2.91**)
Breast	0.5 (2.23)	(**−**4.13, **−**4.63)	0.62 (1.61)	(**−**2.53, 3.78)	**−**0.16 (0.81)		(**−**1.75, 1.41)	**−**0.1(0.65)		(**−**1.39, 1.18)
Genital organs	**5.63 (2.25**)*	**(1.54, 10.37**)	**1.52 (1.62**)*	**(0.34, 6.7**)	1.1 (0.82)		(**−**0.6, 2.6)	1.1(0.66)		(0.11, 2.89)
Other organs	3.27 (2.5)	(**−**1.5, **−**8.29)	1.59 (1.81)	(**−**1.95, 5.15)	1.1(0.92)		(**−**0.76, 2.91)	0.6(0.73)		(**−**0.55, 2.43)
Liver	5.1 (3.04)	(**−**0.75, **−**11.2)	3.01 (2.21)	(**−**1.31, 7.34)	1.63 (1.1)		(**−**0.43, 3.84)	0.06 (0.91)		(**−**1.48, 2.06)
Pancreatic	5.9 (4.4)	(**−**3.02, **−**14.2)	2.2 (3.28)	(**−**4.22, 8.63)	2.3 (1.5)		(**−**0.71, 5.22)	0.9(1.33)		(**−**1.02, 4.18)
Health status of caregivers										
Very good, good	*Ref*									
Fair or poor	**3.61 (1.34**)*	**(0.15, 3.67**)	0.56 (0.64)		**1.34(0.48**)*		(0.41, 3.61)	**1.13 (0.39**)*		(0.34, 1.91)
Constant	**23.04 (2.47**)**	**(18.2, 27.9**)	**11.33 (2.5**)	**(6.43, 16.24**)	**8.62 (1.21**)**		**(6.59, 11.76**)**	**4.47 (0.68**)		**(3.12, 5.81**)
Random effect**	**Estimate** **(SD**)	**95% CI**	**Estimate** **(SD**)	**95% CI**	**Estimate (SD**)		**95% CI**	**Estimate (SD**)		**95% CI**
Between-person effect	8.84 (0.71)	(7.55, 10.35)	2.41 (0.39)	(1.74, 3.32)	2.58 (0.23)		(2.12, 3.04)	2.7 (0.22)		(2.3, 3.17)
Within person effect	5.44 (0.24)	(3.98, 5.94)	3.75 (0.18)	(3.41, 4.12)	1.99 (0.09)		(1.81, 2.2)	1.62 (0.07)		(1.48, 1.77)

† APCA POS score: high scores indicated worse patient status.

* *P*-value < .05.

** *P*-value < .001.

SD *=* standard deviation, SE *=* standard error.

In the full sample (484 observations from 134 patients), physical symptom scores improved significantly by 2.80 points per month (β = −2.79, 95% CI − 3.4 to − 2.2, *P* < .001). A sensitivity analysis restricted to observations from month 6 onward (198 observations from 19 patients) showed that the improvement in physical symptoms remained statistically significant, although the magnitude was smaller (β = −0.44, *P* < .001). Exploratory quadratic analysis on total APCA POS scores demonstrated a significant positive quadratic term (β = 0.28, SE = 0.03, *P* < .001), indicating deceleration of improvement over time. The quadratic model provided a significantly better fit than the linear model (LR χ^2^ (1) = 66.25, *P* < .0001). Detailed results are presented in Table 2.

## 4. Discussion

Palliative care has been implemented in Vietnam since 2011 and is primarily provided at tertiary cancer hospitals. In this model, medical staff mainly focus on assessing and managing the physical symptoms of patients with advanced cancer. The provision of intensive psychological counseling programs or psychological support services for patients and their families remains limited. The roles of physicians and nurses in PC do not encompass intensive psychological counseling, resulting in inadequate support for emotional and psychosocial issues faced by patients and caregivers.

Our study was the first cohort to longitudinally track the health outcomes of cancer patients treated for PC as treatment progressed. This study demonstrated that the patients’ quality of life improved during PC treatment. Patients with higher APCA POS scores on admission showed greater improvement over time. This improvement was consistent from 1 to 9 months. However, from month 10 to the end of the follow-up, the APCA POS scores showed a slight upward trend, suggesting that some symptoms may become more difficult to control. Cancer patients with high APCA POS scores for pain, fatigue, and anorexia at admission tended to show greater improvement over the study period than those with lower APCA POS scores, suggesting that PC is particularly effective in controlling severe physical symptoms, thereby increasing patient comfort and improving daily functioning. Similar results were observed for the emotional and psychosocial domains. Patients with high scores in these domains at admission showed greater improvement during PC. These findings were consistent with those of previous studies showing that patients with severe physical symptoms at the start of PC tended to improve more significantly over the course of treatment.^[22–24]^

An exploratory quadratic mixed-effects analysis confirmed significant nonlinearity in the trajectory of total APCA POS scores. Improvement was most pronounced in the early months and attenuated in the later period, consistent with the slight upward trend observed after month 9 to 10. Sensitivity analyses restricted to later follow-up (from month 6 onward) further supported the robustness of the findings, demonstrating that physical symptoms continued to improve significantly, although at a reduced rate compared with the full sample.

This study, along with previous studies, found that physical symptoms typically improve more quickly, particularly within 2 to 4 weeks of starting PC, than psychological and emotional problems such as anxiety and depression.^[22,23,25,26]^ These differences may arise from the complex nature of psychological issues, which are not only related to the underlying medical condition but also influenced by the patient’s social, family, and mental health factors. These findings are consistent with those of previous studies conducted in several countries, including Vietnam, Taiwan, Chile, North America, and Europe.^[8,22,25,27,28]^ Similarities between these studies include the emphasis on the effectiveness of PC in controlling early physical symptoms, and the need for more specialized psychological support services, such as counseling, group therapy, or active family involvement, to assist patients in overcoming emotional and spiritual challenges.

Similar to most previous studies of terminally ill cancer patients receiving PC, mortality increased significantly as the end-stage approached, accompanied by an escalation in symptom burden.^[29–31]^ This study found that symptoms such as pain and anxiety recurred in the final months of follow-up, possibly attributable to the natural progression of cancer and survival bias, with 76% of patients dying within 1 year. The remaining patients exhibited persistent but progressive deterioration, which may have led to overestimation of the APCA POS scores. The absence of data in the terminal stage may have been due to the MNAR model. This could have biased the results, indicating a significant increase in the severity of patients’ problems.

According to another study conducted in 10 European countries, Australia, and Canada, involving 1739 cancer patients treated with PC for 8 months, patients’ quality of life, including both emotional and physical symptoms, did not change significantly. This may be because all patients in that study were late-stage cancer patients receiving end-of-life care with limited ability to improve symptoms. In contrast, the patients in our study included different stages of the disease, from stable to severe, which may have facilitated faster improvement in physical and emotional symptoms. Furthermore, a retrospective analysis of cancer patients from the time of referral to an outpatient PC center until their first follow-up visit showed an increase in the intensity of physical symptoms after 1 month of follow-up. In our study, physical symptoms significantly reduced after the first month.^[27]^

After adjusting for confounding variables, this study demonstrated that advanced age was significantly associated with improved patient-reported outcome scores in the physical and emotional domains during the study period. These findings are consistent with those of previous studies conducted in Chile and Taiwan, which reported more pronounced physical symptoms in younger patients than in older patients.^[22,27]^ It also added that the presence of a physically and mentally healthy caregiver was significantly correlated with improved patient outcomes across multiple domains, including physical symptom management, emotional health, and communication effectiveness. These observations reinforce the findings of previous studies, which have established a bidirectional relationship between caregiver characteristics and patient health outcomes.^[32,33]^

This study showed that caregiver well-being improved over time. Aspects such as anxiety, need for information, and confidence in caring for the patient improved. These trends were similar to improvements in patient health problems. This result demonstrated a strong association between caregivers and patient well-being. Caregivers play a crucial role in symptom management, treatment adherence, and the provision of emotional and social support. Evidence suggests that cancer patients with physical and emotional distress require more caregiver support. This creates a cumulative burden that reduces the caregivers’ quality of life.^[32,33]^ When caregivers are in good health, they help patients improve their treatment adherence and reduce hospitalization rates. The study also showed that caregivers’ APCA POS scores improved over time. However, it recommends additional measures, such as training in illness management skills and psychological support services for caregivers.^[34,35]^

The results also showed that cancer diagnosis affected patients’ ability to improve their health outcomes. Patients diagnosed with respiratory, head and neck, or genitourinary cancers showed a significantly increased severity of physical symptoms and psychosocial problems, especially in the last months of follow-up. Patients diagnosed with genitourinary cancers had higher total APCA POS scores than those diagnosed with gastrointestinal cancers. Patients with genitourinary cancers are deeply affected by the disease and side effects of treatment, leading to physical changes and an impact on psychosocial, emotional, and quality of life.^[36]^ This may hinder the use of conventional and supportive approaches to improve patients’ psychosocial condition. Similarly, patients with head, neck, and respiratory cancers had higher APCA POS scores for emotional and psychosocial problems than patients diagnosed with gastrointestinal cancers. This difference may be due to the invasive nature of head and neck and respiratory cancers, which can cause prolonged pain and affect social communication, resulting in a greater psychological burden. Patients with head and neck cancer not only have to endure a potentially fatal disease but also face inevitable changes in appearance and a decline in fundamental functions such as swallowing, chewing, breathing, and communication. Therefore, patients may experience severe psychological problems, such as anxiety, depression, feelings of inferiority, and a sense of meaninglessness in life.^[37–39]^ In patients with respiratory cancers, depression and anxiety are the two most common psychiatric problems and are significantly associated with shortness of breath, panic attacks, and reduced survival.^[40]^ A study at the Massachusetts General Hospital Cancer Center compared the quality of life of cancer patients who received PC and usual care and found significant differences in the improvement of quality of life associated with cancer diagnosis. In particular, patients with lung cancer who received PC had an improved quality of life and reduced depressive symptoms at weeks 12 and 24. In patients with gastrointestinal cancer, both groups reported improvements in quality of life and mood at week 12. Patients who received PC were more likely to discuss their end-of-life wishes with their treating physicians, especially as they approached the end of life.^[41]^ These results suggest that cancer diagnosis can have a significant impact on improving physical and psychosocial problems for patients.

This study was strengthened by its longitudinal design, which enabled the identification of long-term changes in health outcomes and the psychological and social challenges faced by cancer patients. This is the first study to provide evidence of changes in the quality of life of patients with cancer receiving PC over time in Vietnam. The highlights of this study are the relatively large sample size and the use of the APCA POS scale, a widely recognized tool in the field of PC, to systematically measure health outcomes, which helps ensure consistency and increases comparability with studies worldwide.

However, this study had several limitations that should be considered. First, the recruitment process may have excluded patients with poor health who were unable to consent to participate, leading to an underestimation of health problems in those most in need of PC. Second, reliance on telephone follow-up may have introduced reporting bias, as patients or surrogates may have underreported symptoms because they were not directly assessed. Finally, a limitation of the data analysis in this study was the assumption that missing data during patient follow-up were MAR, and that an LMM was used to examine differences in APCA POS scores over time and between patients. Therefore, this limitation underscores the need for future studies to employ more advanced methods, such as multiple imputation under the MNAR assumption or a joint survival model, to mitigate potential bias and enhance the accuracy of PC outcome interpretation.

Furthermore, although sensitivity analyses restricted to month 6 onward and exploratory quadratic models supported the robustness and nonlinearity of the findings, the primary analyses assumed linear time effects and MAR. Future studies with larger sample sizes should consider joint survival models or pattern-mixture approaches to more comprehensively address potential MNAR mechanisms associated with high mortality.

## 5. Conclusions

This is the first longitudinal study in Vietnam to repeatedly describe changes in physical symptoms, emotional problems, and social functioning in patients with cancer receiving long-term PC. These findings suggest that PC can improve the health outcomes of cancer patients in the early stages, with the most significant improvements observed in physical symptoms, particularly within the first month. Changes in patients’ health outcomes were influenced by age, cancer diagnosis, and caregivers’ health status. These findings highlight the vital role of providing early PC and psychological support to patients and caregivers.

## Acknowledgments

We thank the Evaluation of the Six WHO Palliative Care Demonstration Sites Project of the Cicely Saunders Institute of Palliative Care Policy and Rehabilitation at King’s College London and the leaders of Ho Chi Minh City Oncology Hospital for their suggestions, approval, and facilitation of this study.

## Author contributions

**Conceptualization:** Thuy Mai, Dung Do, Cheng-Pei Lin, Richard Harding.

**Data curation:** Thuy Mai, Ninh Uong, Cheng-Pei Lin.

**Investigation:** Thuy Mai.

**Methodology:** Thuy Mai, Oanh Trinh, Dung Do, Cheng-Pei Lin, Richard Harding.

**Project administration:** Thuy Mai.

**Visualization:** Thuy Mai.

**Supervision:** Oanh Trinh, Richard Harding.

**Validation:** Oanh Trinh.

**Software:** Dung Do, Ninh Uong.

**Formal analysis:** Ninh Uong.

**Writing – original draft:** Thuy Mai, Oanh Trinh, Dung Do, Cheng-Pei Lin.

**Writing – review & editing:** Thuy Mai, Oanh Trinh, Dung Do, Ninh Uong, Cheng-Pei Lin.
